# Esophageal Granular Cell Tumor Associated With Eosinophilic Gastrointestinal Disease

**DOI:** 10.14309/crj.0000000000001720

**Published:** 2025-06-04

**Authors:** Cathy Zheng, Robert Mowery, Sareena Ali, Camilla Allen, Ryan T. Hoff

**Affiliations:** 1 Department of Medicine, University of Miami Miller School of Medicine, Miami, FL; 2 Elson S. Floyd College of Medicine, Washington State University, Spokane, WA; 3 Department of Medicine, Advocate Lutheran General Hospital, Park Ridge, IL; 4 PeaceHealth Medical Group, Vancouver, WA

**Keywords:** granular cell tumor, esophagus, eosinophilic esophagitis, eosinophilic gastrointestinal disease

## Abstract

Granular cell tumors (GCTs) are typically benign lesions that can occur in the gastrointestinal tract. Eosinophilic gastrointestinal disease, including eosinophilic esophagitis (EoE), is an immune-mediated condition characterized by eosinophil-predominant inflammation in the gastrointestinal tract. We review previously reported cases of EoE associated with GCTs. To our knowledge, we present the first case of esophageal GCT in a patient with a long history of eosinophilic gastrointestinal disease and likely several years of active EoE before esophageal GCT diagnosis.

## INTRODUCTION

Granular cell tumors (GCTs) are lesions thought to be of Schwann cell origin that occur in the breast, skin, tongue, and gastrointestinal tract.^1^ However, they are relatively rare in the esophagus, accounting for about 1% of all GCTs and 1.2% of benign esophageal tumors, with an incidence of approximately 0.033% in esophagogastroduodenoscopies (EGDs).^2,3^ On endoscopy, esophageal GCTs (eGCTs) appear as sessile, yellow-white lesions covered by normal-appearing mucosa.^4,5^ Most eGCTs are located in the distal esophagus (65%), with fewer found in the mid (20%) and proximal (15%) esophagus.^1^ Although typically asymptomatic and discovered incidentally, larger lesions may cause dysphagia, chest pain, cough, nausea, and gastroesophageal reflux.^6^ eGCTs are more common in men, and the mean age of diagnosis is 45 years.^6,7^ Most GCTs are benign; only about 1%–2% are reported to be malignant.^6^

Eosinophilic esophagitis (EoE) is a chronic, immune/antigen-mediated disease characterized histologically by eosinophil-predominant inflammation that causes symptoms of esophageal dysfunction.^8^ There are 3 diagnostic criteria for EoE: (i) symptoms of esophageal dysfunction; (ii) esophageal biopsy with at least 15 eosinophils per high-power field (eos/hpf); and (iii) evaluation of the differential diagnosis of EoE. There is a possible association between EoE and GCTs. Here, we report the case of an eGCT diagnosed in a patient with a long history of eosinophilic gastrointestinal disease (EGID).

## CASE REPORT

**Figure 1.**
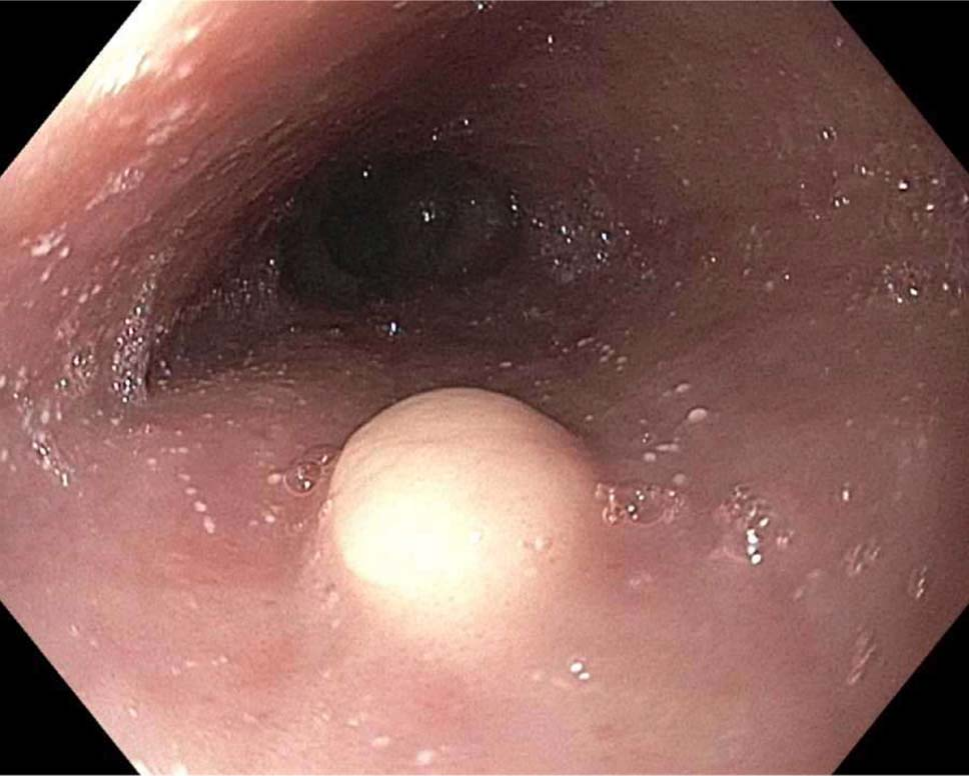
Image from initial upper endoscopy showing esophageal granular cell tumor at 31 cm from the incisors, which appears sessile and white. Nearby mucosa shows esophageal exudates (eosinophilic microabscesses).

**Figure 2.**
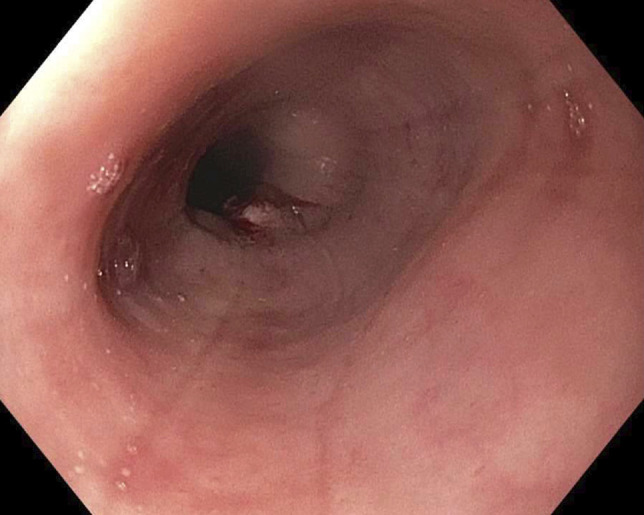
Image from initial upper endoscopy showing longitudinal furrows. Eosinophilic esophagitis endoscopic reference score: E1 R0 Ex1 F1 S0.

**Figure 3.**
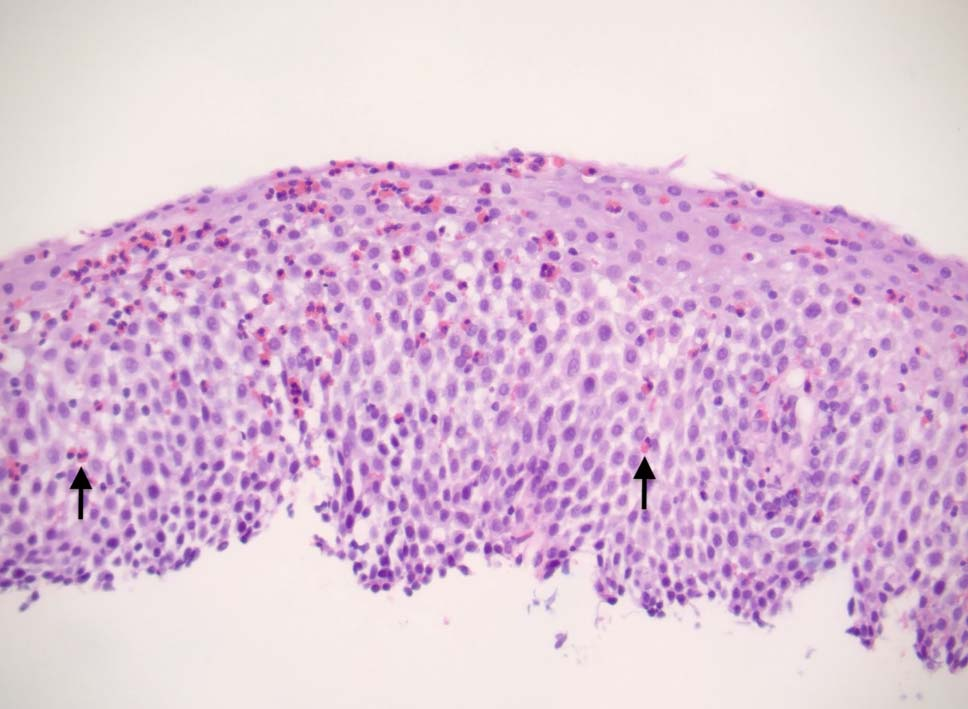
Eosinophilic esophagitis. Note the red eosinophils infiltrating the epithelium (black arrows). Magnification 200×.

**Figure 4.**
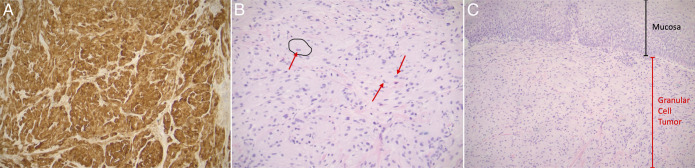
(A) Granular cell tumor positive for S-100 immunohistochemical stain with 100× magnification. (B) Granular cell tumor with epithelioid features characterized by large cells with abundant pink, granular cytoplasm due to numerous lysosomes. Black outline shows cytoplasm of GCT cell. Red arrows show GCT nuclei. 200× magnification. (C) Granular cell tumor showing overlying squamous mucosa. Note that the GCT (red bar) does not invade into the mucosa (black bar). Magnification 100×. GCT, granular cell tumors.

A 33-year-old woman presented with chronic regurgitation and progressive intermittent dysphagia to solid foods. Approximately 12 years prior, she was diagnosed with EGID via EGD and completed a short course of intravenous corticosteroids but had since remained off therapy. Repeat EGD was performed given her current symptoms, revealing esophageal edema, longitudinal furrows, and exudates (EoE endoscopic reference score of 3). Also present was duodenal erythema and a single white sessile mucosal nodule in the mid-esophagus at 31 cm (Figures 1 and 2). Biopsies showed >50 eos/hpf in both the proximal and distal esophagus, and up to 60 eos/hpf in gastric lamina propria. Duodenal biopsies showed >100 eos/hpf, with foveolar metaplasia and villous blunting in eosinophil rich areas (Figure 3). Biopsy of the mid-esophageal lesion showed a GCT (Figure 4). Laboratory workup including blood counts, chemistries, inflammatory markers, and iron studies were all normal. Stool was negative for ova and parasites.

The patient started a 6-food elimination diet (avoiding animal milk, wheat, egg, soy, nuts, and fish/shellfish). She reported symptom improvement after 8 weeks but still had occasional regurgitation. Follow-up EGD at 8 weeks redemonstrated a 5-mm nodule in the mid-esophagus, which was completely removed via endoscopic mucosal resection. EoE endoscopic reference score decreased to 2. Pathology showed a GCT without any features associated with aggressive behavior. Gastric and duodenal biopsies showed resolution of previously prominent EGID, without eosinophilia. Esophageal biopsies showed improved EoE, with up to 5 eos/hpf in the distal esophagus and up to 20 eos/hpf in the mid-esophagus. Recommendations included starting omeprazole 20 mg daily and reintroducing soy.

At the second follow-up EGD 4 weeks later, the patient's symptoms remained overall well-controlled. The patient did not start omeprazole. EGD showed grossly normal esophagus and stomach, and duodenal erythema. Gastric and duodenal biopsies showed no significant eosinophilic inflammation, whereas proximal and distal esophageal biopsies showed up to 12 eos/hpf, overall consistent with continued remission. Recommendations were to reintroduce wheat and repeat EGD in 6–8 weeks to assess response.

## DISCUSSION

The association between eGCT and EoE remains a topic of interest, with mixed evidence regarding their connection. To our knowledge, there have been 23 documented cases of confirmed EoE associated with eGCT (Table 1), excluding a case series by Turner et al due to limited details and uncertainty in the diagnosis of EoE.^9^ Including the present case, 42% of patients were female (10) and 58% were male (14). There were 9 pediatric cases and 15 adults. The average age was 25.9 years. Dysphagia was the most common presenting symptom, occurring in 54% of cases. Data on race/ethnicity were limited. In 58% of cases, eGCT was diagnosed concurrently with or before EoE. In 8.3% of cases, eGCT was discovered in adult patients with recent diagnoses of EoE. Approximately 13% of patients had 2 or more years of EoE before eGCT was diagnosed; however, these were pediatric cases. Our case is unique as it presents an eGCT in an adult patient with long-standing active EoE.

**Table 1.** Case reports of eosinophilic esophagitis associated with an esophageal granular cell tumor

Case number	Age	Gender/sex	Race/ethnicity	Symptom(s)	Duration of EoE/EGID before eGCT	Eosinophil count	References
1	41	M	NR	Dysphagia	EoE onset 2 y after eGCT	>24	Lucendo et al^10^
2	50	F	White	Dysphagia	Simultaneous	75	Nojkov et al^11^
3	36	M	African American	Pyrosis	Simultaneous	>20	Nojkov et al^11^
4	15	M	White	Dysphagia, prior food impactions	Simultaneous	>20	Nojkov et al^11^
5	29	F	African American	Dysphagia, globus sensation	Simultaneous	>20	Nojkov et al^11^
6	38	F	White	Dysphagia, pyrosis	Simultaneous	>20	Nojkov et al^11^
7	35	M	NR	Food impactions	Simultaneous	67	Riffle et al^12^
8	39	F	NR	Dysphagia	Simultaneous	62	Riffle et al^12^
9	37	M	NR	Dysphagia	Simultaneous	68	Riffle et al^12^
10	32	M	NR	Food impactions, heartburn, abdominal pain	Simultaneous	38	Riffle et al^12^
11	14	F	NR	Dysphagia, heartburn	Simultaneous	24	Riffle et al^12^
12	15	F	NR	Disordered feeding	Simultaneous	34	Riffle et al^12^
13	25	M	NR	None	“Recent”	NR	Abughofah et al^13^
14	3	M	NR	NR	NR	NR	Malik et al^14^
15	4.5	M	NR	NR	NR	NR	Malik et al^14^
16	4	M	NR	NR	NR	NR	Malik et al^14^
17	16	F	NR	Dysphagia	2 years	Not reported (mild)	Mohammad et al^15^
18	9	M	NR	Asymptomatic	7 y	0	Kuhn et al^16^
19	16	M	NR	Asymptomatic	2 y	0	Kuhn et al^16^
20	33	F	NR	Dysphagia	NR	82	Reddi et al^17^
21	25	F	NR	Dysphagia	Simultaneous	Innumerable	Stone et al^18^
22	38	M	NR	NR	12 wk	NR	Stone et al^18^
23	33	M	NR	Dysphagia	Simultaneous	NR	Stone et al^18^
24	33	F	White	Dysphagia, regurgitation	12 y	>50	Current case report

EoE, eosinophilic esophagitis; EGID, eosinophilic gastrointestinal disease; eGCTs, esophageal GCTs; GCT, granular cell tumors; NR, not reported.

Several reports have proposed potential pathophysiological mechanisms linking EoE and eGCT. Nojkov et al observed that patients with concurrent EoE and eGCT tended to have larger eGCT nodules compared to those with eGCT alone, suggesting that larger eGCTs might stimulate EoE development.^11^ Hypothesized mechanisms include the possibility that eGCTs may induce eosinophil mucosal infiltration through allergen production or cytokine stimulation. In addition, there could be a shared genetic pathway between the 2 disorders. Riffle et al noted that GCTs have been associated with tissue inflammation in mastectomy scars and chronic appendicitis, supporting the theory that GCTs are reactive in nature.^12,19^ The presence of intratumoral eosinophilia in many GCTs further supports these hypotheses.^20^

The temporal relationship between EoE and eGCT is also noteworthy. On average, the reported cases of eGCTs in patients with EoE were diagnosed about 19 years earlier than in those without EoE, suggesting that eosinophilic inflammation might drive the earlier development of eGCTs. Despite these findings, the evidence linking EoE and eGCTs is somewhat mixed, with a retrospective multicenter study by Reddi et al showing no association.^17^ Therefore, further investigation is necessary to clarify the relationship between these conditions and underlying mechanisms. Recognizing a potential connection between EoE and eGCTs could improve the management of patients with these conditions. Both conditions should be included in the differential diagnosis for dysphagia, especially for younger patients. When an eGCT is diagnosed endoscopically, it is important to evaluate the surrounding tissue for evidence of EoE. In addition, clinicians should recognize eGCT as a rare but potential long-term complication arising from chronic EoE inflammation.

## DISCLOSURES

Author contributions: C. Zheng reviewed the literature and wrote the initial and revisional drafts. R. Mowery and S. Ali performed literature reviews and provided critical edits to the manuscript. C. Allen evaluated pathology slides, provided Figures 3 and 4, and edited sections pertinent to histologic findings. R. Hoff is the article guarantor and is responsible for concept and design, critical review of the manuscript for important intellectual content, and clinical care of the patient.

Financial disclosure: None to report.

Previous presentation: ACG Annual Scientific Meeting, October 28, 2024, Philadelphia, PA.

Informed consent was obtained for this case report.
